# Supplementary material for: Lead-Free Copper-Based Perovskite Nanonets for Deep Ultraviolet Photodetectors with High Stability and Better Performance
Source: Nanomaterials (Basel). 2022 Sep 20;12(19):3264. doi: 10.3390/nano12193264 (PMC9565817; doi:10.3390/nano12193264)
Supplement: Supplementary file 1 [file nanomaterials-12-03264-s001.zip › nanomaterials-1914385-supplementary.pdf]

Supporting Information

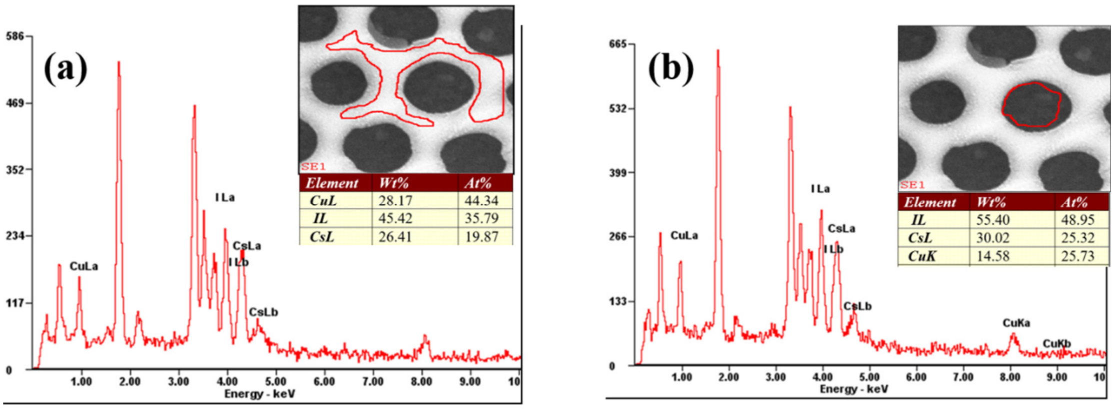

Figure S1. Energy dispersive X-ray spectroscopy (EDS) and the corresponding scanning electron microscopy images of $Cs_3Cu_2I_5$ nanoholes raised and depressed parts (a, b)

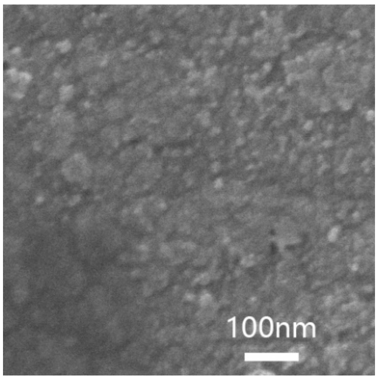

Figure S2. SEM of $Cs_3Cu_2I_5$ compact film prepared by the traditional spin-coating.

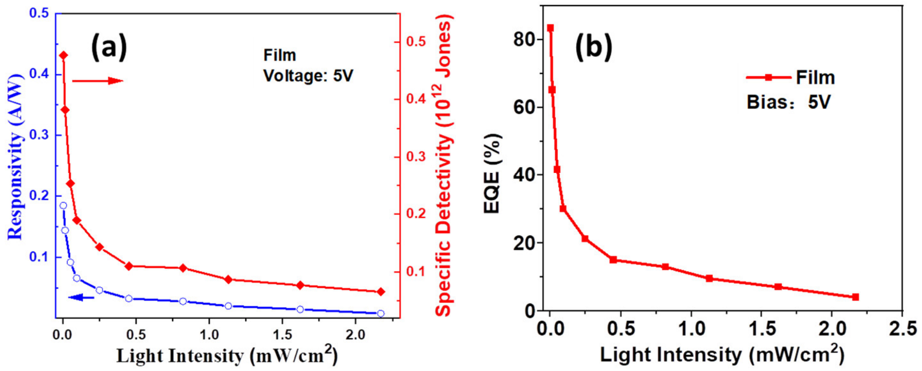

Figure S3. (a) Responsivity and detectivity of the compact film photodetector versus the light density. The biggest responsivity is 0.185A W^{-1}, the highest detectivity is 0.48×10^{12} Jones. (b) The EQE of the compact film photodetector versus the light density. The highest EQE is 85%.

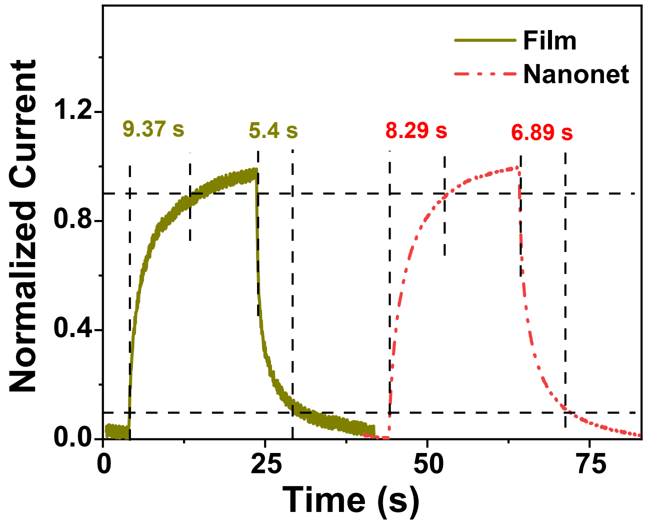

Figure S4. The normalized current of film PD or nanonet PD at an off-on-off circle by using a chopper. The rise time and decay time were estimated at 90% or 10% maximal photo current.

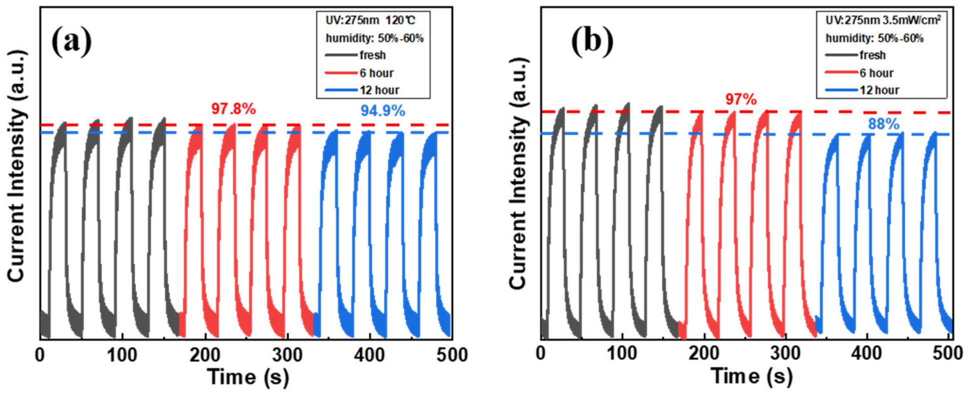

Figure S5. The normalized I-t curves of the nanonet photodetector freshly-fabricated or treated at different environments. (a) under thermal environment at temperature of 120 ℃ for 6 h and 12 h. (b) under 275nm, 3.5 mW/cm^2 strong ultraviolet light illumination. After treatment, I-t curve of PD was tested under the same condition by using a chopper to turn on or turn off the photo.
